# Putrescine delays postovulatory aging of mouse oocytes by upregulating PDK4 expression and improving mitochondrial activity

**DOI:** 10.18632/aging.101699

**Published:** 2018-12-16

**Authors:** Wendan Xu, Lingjun Li, Jingwen Sun, Songyue Zhu, Zhengjie Yan, Li Gao, Chao Gao, Yugui Cui, Caiping Mao

**Affiliations:** 1 Reproductive Medicine Center, First Affiliated Hospital of Soochow University, Jiangsu Sheng, China; 2 State Key Laboratory of Reproductive Medicine, Clinical Center of Reproductive Medicine, First Affiliated Hospital, Nanjing Medical University, Nanjing, China

**Keywords:** oocyte, postovulatory aging, PDK4, mitochondria, reactive oxygen species

## Abstract

If fertilization does not occur for a prolonged period *in vivo* or *in vitro*, the postovulatory oocytes will deteriorate, which called the postovulatory aging. This process disrupts the developmental competence. In the present study, we showed that the reactive oxygen species (ROS) was accumulated in oocytes during the postovulatory aging. ROS inhibited Sirt1 expression, and then increased oxidative stress by downregulating the intracellular Sirt1-FOXO3a-SOD2 axis. Moreover, the inhibited Sirt1 expression was related to the decreased mitochondrial function and the lowered level of autophagy. The mitochondrial-related apoptosis was increased by inhibiting the AKT and ERK1/2 pathways, due to the accumulation of ROS in the postovulatory oocytes. The mitochondrial pyruvate dehydrogenase kinase-4 (PDK4) can reduce ROS by inhibiting the tricarboxylic acid (TAC) cycle. We found that PDK4 was significantly decreased in the postovulatory aging oocytes. Putrescine, one of the abundant biogenic amines, ameliorated the effects of ROS and therefore improved the quality of the postovulatory aging oocytes by increasing the expression of PDK4. When PDK4 was downregulated using siRNAs, the effects of putrescine were significantly receded. We concluded that putrescine delayed the aging process of postovulatory oocytes by upregulating PDK4 expression and improving mitochondrial activity.

## Introduction

The postovulatory oocyte is generally arrested at the metaphase stage of the second meiosis (MII), where it awaits for fertilization. The early embryo will be successfully developed if fertilization occurs within 10 h of ovulation [1]. If fertilization does not occur during this postovulatory period, the oocyte will progressively undergo a time-dependent deterioration in quality, which is called “postovulatory aging” [2]. Numerous studies have shown that the postovulatory aging is associated with a range of defects, including abnormalities in the structures of the mitochondria [3], meiotic spindle and chromosome organization [4]. The postovulatory aging is also accompanied by diverse biochemical and molecular changes, such as the increased production of reactive oxygen species (ROS), the decreased expression of the anti-apoptotic factor Bcl-2, the hyperactivation of caspase-3 and the potential changes in epigenetic modification [5–7]. These aging-related changes at the morphological, cellular and molecular levels will impair the fertilization and subsequent embryo development.

The postovulatory aging increasingly occurs in the *in vitro* culture and treatment of the oocytes of assisted reproductive technologies (ART). ARTs have been widely used in the treatment of infertility, in which gametes are maintained under *in vitro* conditions for a prolonged period. This prolonged period and the *in vitro* condition might compromise the subsequent fertilization and embryo development, which could be over the effects of the *in vivo* procedure of postovulatory aging. For example, many studies showed that the prolonged *in vitro* culture of a matured oocyte, prior to *in vitro* fertilization (IVF) or an intracytoplasmic sperm injection (ICSI), affects the quality of both human and mouse oocytes [8,9]. It is therefore important to elucidate the changes in the cellular functions and underlying mechanisms of those affected oocytes undergoing the *in vivo* and *in vitro* postovulatory aging, so that the correct approach to delay postovulatory aging can be developed.

Polyamines, including putrescine, spermine and spermidine, are produced from amino acids (e.g., ornithine, arginine [Arg] and proline) in mammalian cells, playing multiple roles in maintaining cellular activity and signal transduction [10,11]. For example, studies demonstrated that polyamines may alleviate the oxidative stress in stressed plants by regulating the antioxidant systems along with the decreased ROS production [12,13]. It has recently been demonstrated that putrescine also exerts antioxidant, anti-apoptotic, and anti-inflammatory effects and protects various types of cells or tissues against oxidative stress [14]. Moreover, recent studies have also shown that putrescine improves the nuclear maturation in mouse oocytes and subsequent embryo development in older mice [15]. However, the detailed mechanism and potential application of putrescine in reproduction have not yet been elucidated.

Mitochondrial pyruvate dehydrogenase kinase 4 (PDK4) inactivates the pyruvate dehydrogenase complex by phosphorylating pyruvate dehydrogenase (PDH), thereby dictating the conversion of pyruvate to acetylcoenzyme A (Acetyl-CoA) [16]. The primary research focused on the PDK4-related metabolic function over the past decade; however, emerging evidence suggests that the additional roles of PDK4 include promoting tumorigenesis, antioxidant formation [17], and anti-apoptosis in the liver [18,19]. It was discovered that the pro-survival pathway was switched to the proapoptosis under a PDK4-deficient condition. This PDK4 deficiency triggered hepatic apoptosis concomitantly with the increased numbers of aberrant mitochondria and production of ROS, the sustained activation of c-JunN-terminal Kinase (JNK), and the increased level of reduced glutathione (GSH) [20]. However, the roles of PDK4 in oocytes, especially in the postovulatory aging of oocytes, are currently unknown.

In the present study, we investigated whether putrescine can protect oocytes from the postovulatory aging, and we also investigated the underlying cellular and molecular mechanisms of this protection, so as to explore the potential application of putrescine as a protective factor or an antioxidant in the *in vitro* culture of oocytes.

## RESULTS

### Putrescine decreased the apoptotic index during the postovulatory aging of oocytes and alleviated morphological changes

**Figure 1:**
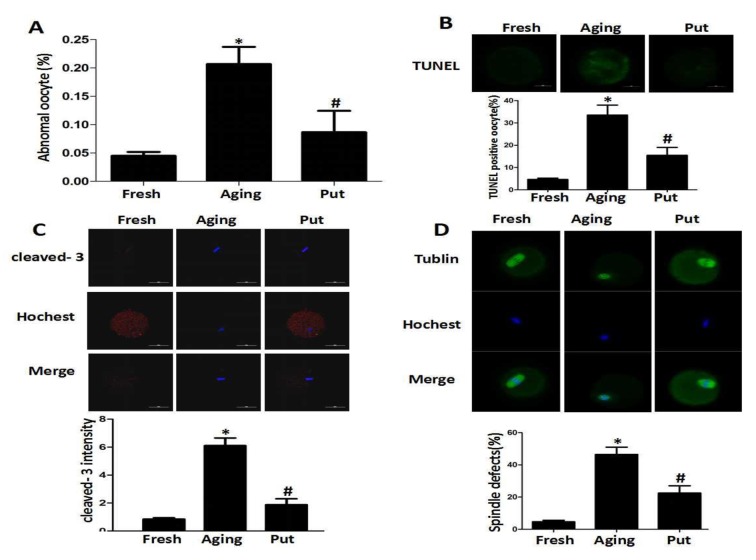
**Putrescine decreased the apoptosis index and alleviated morphological changes during the postovulatory aging of oocytes.** (**A**) The morphological defects of oocytes during the postovulatory aging process. The proportion of abnormal oocytes was increased in the oocytes undergoing postovulatory aging. When the MII oocytes were exposed to 0.5 mM of putrescine, the rate of morphological defects was significantly lowered when compared with the aging oocytes. (**B**) The level of apoptosis in the oocytes during postovulatory aging. TUNEL analysis confirmed the increased apoptosis during the postovulatory aging of oocytes. The apoptosis was significantly inhibited by the 0.5 mM of putrescine that was added to the *in vitro* culture medium. (**C**) The activated caspase 3 showed the increased level of apoptosis in the oocytes during postovulatory aging. The level of cleaved caspase 3 was significantly increased in the aging oocytes. Putrescine significantly inhibited the activation of caspase 3 in the aging oocytes. (**D**) The morphological observation of spindles. In the aging oocytes, the spindles became elongated, the microtubules were gradually lost from the spindle, and the aberrant chromosomal alignment was increased. The proportion of abnormal spindles was significantly decreased in the putrescine-treated group. Put, putrescine. Compared with the fresh MII oocytes, *p<0.05; compared with the aging oocytes, # *p*<0.05.

After 24 h of *in vitro* culture (as the model of postovulatory aging), the aged oocytes in the control group showed various morphological defects, including degeneration, fragmentation and parthenogenetic activation when compared with the freshly ovulated oocytes. To explore the potential involvement of putrescine in the aging-induced morphological changes, we treated the MII oocytes cultured *in vitro* with 0 or 0.5 mM putrescine (Figure 1). When the MII oocytes were exposed to 0.5 mM putrescine in the medium, the number of morphological defects was significantly lowered when compared with the aged oocytes (Figure 1A). TUNEL analysis confirmed the increased level of apoptosis during the postovulatory aging of oocytes. Since the postovulatory aging process culminates in apoptosis [21], it is possible that putrescine acts as a protective agent to reduce the apoptotic level of aging oocytes. The apoptotic index was significantly decreased following putrescine treatment (Figure 1B). Moreover, we observed a significant rise in the level of caspase 3 activation in the aging oocytes. However, the level of caspase 3 activation was significantly decreased following putrescine treatment after 24 h of *in vitro* culture (Figure 1C). The MII oocytes were exposed to putrescine (or not) for 24 h and then immunolabeled for the analysis of spindle organization. As shown in Figure 1D, the fresh oocytes displayed the morphology of typical barrel-shaped spindles. However, the spindles in the aging oocytes became abnormal, and the microtubules were gradually lost from the spindle, while the aberrant chromosome alignment was increased. The analysis of the MII oocytes after the 24 h aging period showed that the proportion of abnormal spindles in the putrescine-treated group was significantly lower than that in the control group (Figure 1D).

### ROS was accumulated in the aging oocytes, and putrescine decreased ROS by Sirt1-FOXO3a-SOD2 axis

**Figure 2:**
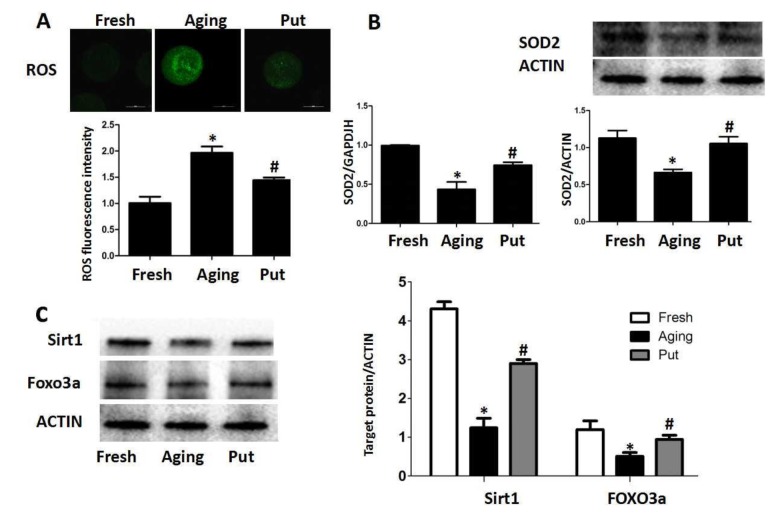
**ROS accumulated in the aging oocytes and putrescine decreased ROS through the Sirt1-FOXO3a-SOD2 axis. (A**) The accumulation of ROS in the oocytes during postovulatory aging. The level of ROS was significantly decreased in the putrescine-treated group. (**B**) The mRNA and protein expressions of SOD2. Both the mRNA and protein expressions of SOD2 were significantly decreased in the aging oocytes, while the expression of SOD2 was partially rescued by putrescine treatment. (**C**) The Sirt1-FOXO3a-SOD2 axis in the aging oocytes. The expressions of Sirt1, FOXO3a and SOD2 protein were significantly decreased in the postovulatory aging oocytes. Putrescine partially rescued the effects on the expressions of Sirt1, FOXO3a and SOD2 protein. Put, putrescine. Compared with the fresh MII oocytes, *p<0.05; compared with the aging oocytes, # *p*<0.05.

Oxidative stress has been considered as a key mechanism of cellular aging [22,23]. Therefore, we measured the intracellular ROS level after 24 h of *in vitro* culture of the postovulatory oocytes. We found that ROS was accumulated in the oocytes during the postovulatory aging process. Putrescine treatment significantly decreased the ROS accumulation (Figure 2A). The expressions of SOD2 at both mRNA and protein levels were significantly decreased in the aging oocytes, while those expressions were partially rescued by putrescine treatment (Figure 2B). The Sirt1-FOXO3a-SOD2 pathway was reported to inhibit oxidative stress in embryonic cells [24,25]. To determine whether putrescine prevented the ROS accumulation in aging oocytes by this pathway, the postovulatory oocytes were treated with or without putrescine for 24 h of *in vitro* culture, and the expressions of Sirt1 and FOXO3a were then assessed. The protein expressions of Sirt1 and FOXO3a were significantly decreased in the postovulatory aging oocytes. Putrescine treatment partially rescued the expressions of Sirt1 and FOXO3a (Figure 2C).

### Functional degeneration of mitochondria in the aging oocytes

**Figure 3:**
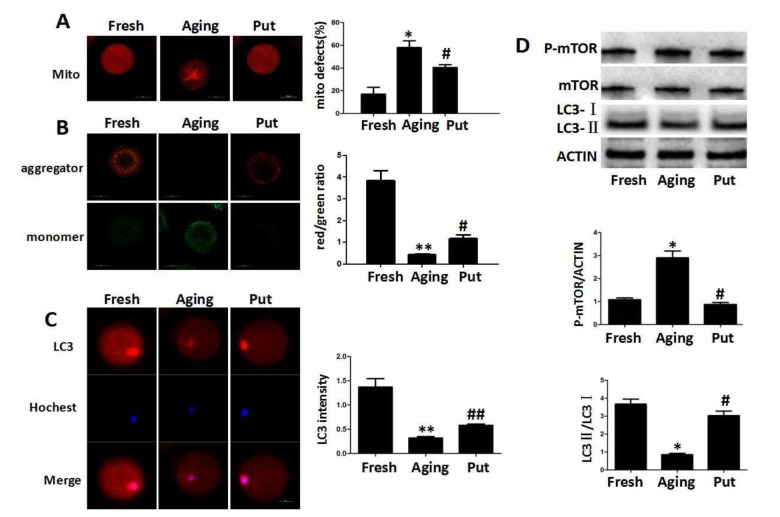
**Functional degeneration of mitochondria in the aging oocytes.** (**A**) The distribution of mitochondria in the cytoplasm. The even distribution of the mitochondria generally suggested a high function, while the aggregated mitochondria always showed functional degeneration. In the fresh MII oocytes, most of the mitochondria were evenly distributed in the cytoplasm. The proportion of aggregated mitochondria was significantly increased in those aging oocytes. However, this number was significantly decreased by putrescine treatment. (**B**) The mitochondrial membrane potential (MMP) was tested by the JC-1 assay to evaluate the mitochondrial activity. The ratio of red/green fluorescence intensity was used as the MMP index. The MMP index was significantly reduced in the aging oocytes when compared with the fresh MII oocytes. Putrescine treatment significantly enhanced the MMP of the aging oocytes. (**C**) The expression level of total LC3 in oocytes during postovulatory aging. The autophagy level was evaluated with total LC3 as an indicator. The autophagy in the fresh MII oocytes was at a reasonable level. The autophagy was significantly decreased in oocytes during postovulatory aging. The autophagy level was partially rescued by putrescine in the aging oocytes. (**D**) mTOR and LC3 transform as autophagy-related factors. The expression of mTOR was increased and the LC3-II/LC3-I ratio decreased in the aging oocytes. These effects were partially rescued by putrescine treatment in the aging oocytes. Mito, mitochondria. Put, putrescine. Compared with the fresh MII oocytes, *p<0.05, ** *p*<0.01; compared with the aging oocytes, # *p*<0.05, ## *p*<0.01.

The decreased expression of Sirt1 has recently been found to be associated with the deterioration of oocyte quality by regulating the mitochondrial function during the postovulatory aging. The even distribution of mitochondria generally suggested a high mitochondrial activity, whereas the aggregated distribution of mitochondria always showed a functional degeneration [25]. In the fresh MII oocytes, most of the mitochondria were evenly distributed in the cytoplasm. The proportion of aggregated mitochondria was significantly increased in those aging oocytes. However, this proportion was significantly decreased by putrescine treatment (Figure 3A). The mitochondria membrane potential (MMP) was also tested by the JC-1 assay to evaluate the mitochondrial activity in our study. The ratio of red/green fluorescence intensity was used as the MMP index (Figure 3B). The MMP index was significantly reduced in the aging oocytes when compared with the fresh MII oocytes. Putrescine significantly increased the MMP index of those aging oocytes. Since Sirt1 induces autophagy by decreasing the activity of mTOR [26], it was of interesting to examine whether putrescine preserves autophagy during the postovulatory aging (Figure 3C). The autophagy level was evaluated using the total LC3 level as an indicator. We found that the autophagy was significantly decreased after 24 h of *in vitro* culture. However, the level of autophagy was restored when the aging oocytes were treated with putrescine (Figure 3C). In addition, the LC3-II/LC3-I ratio was significantly decreased, and the expression level of p-mTOR was increased in the aging oocytes, while putrescine treatment partially prevented those effects during the postovulatory aging of oocytes (Figure 3D).

### The apoptosis-related pathways in the aging oocytes and the effect of putrescine

**Figure 4:**
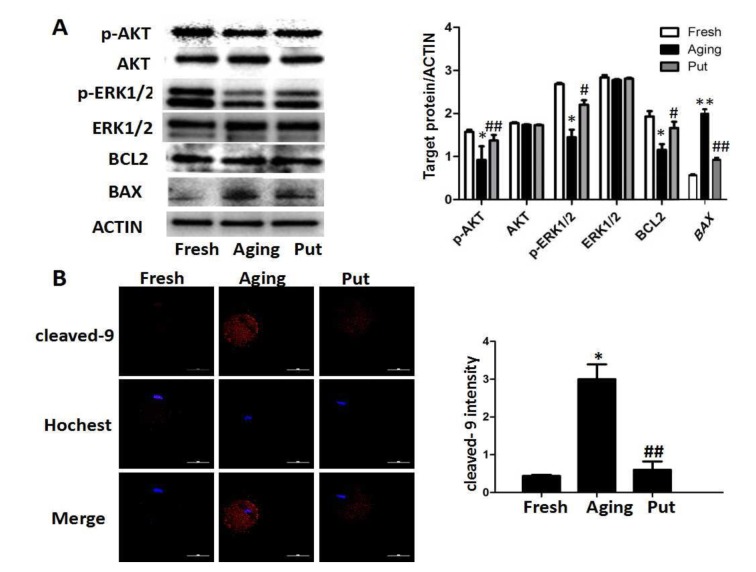
**The apoptosis-related pathways in the aging oocytes and the effect of putrescine.** (**A**) The phosphorylation levels of the AKT and ERK1/2 proteins and the expressions of BCL2 and BAX in the aging oocytes. The levels of p-AKT and p-ERK1/2 were significantly decreased in the oocytes during postovulatory aging, although the total level of AKT or ERK1/2 was not changed. The level of BCL2 was significantly decreased, and the level of BAX increased, as two subsequent factors of the AKT and ERK pathways. Putrescine rescued those effects in the aging oocytes. (**B**) The expression of caspase 9 as a key factor of the mitochondria-related apoptosis pathways. The level of cleaved caspase 9 was significantly increased in the aging oocytes. Putrescine significantly inhibited this incremental rise. Compared with the fresh MII oocytes, *p<0.05, ** *p*<0.01; compared with the aging oocytes, # *p*<0.05, ## *p*<0.01.

An immunoblot analysis revealed that the levels of p-AKT, p-ERK1/2 and BCL-2 were significantly decreased and the level of BAX was increased during postovulatory aging. However, putrescine treatment significantly reduced those downregulations of p-AKT, p-ERK1/2 and BCL-2 and the upregulation of BAX (Figure 4A). Moreover, a significant rise in the level of caspase 9 activation was found in the aging oocytes; the level of caspase 9 activation was significantly reduced after 24 h of putrescine treatment in the *in vitro* culture (Figure 4B).

### Effects of putrescine on PDK4 expression and oxidative stress

**Figure 5:**
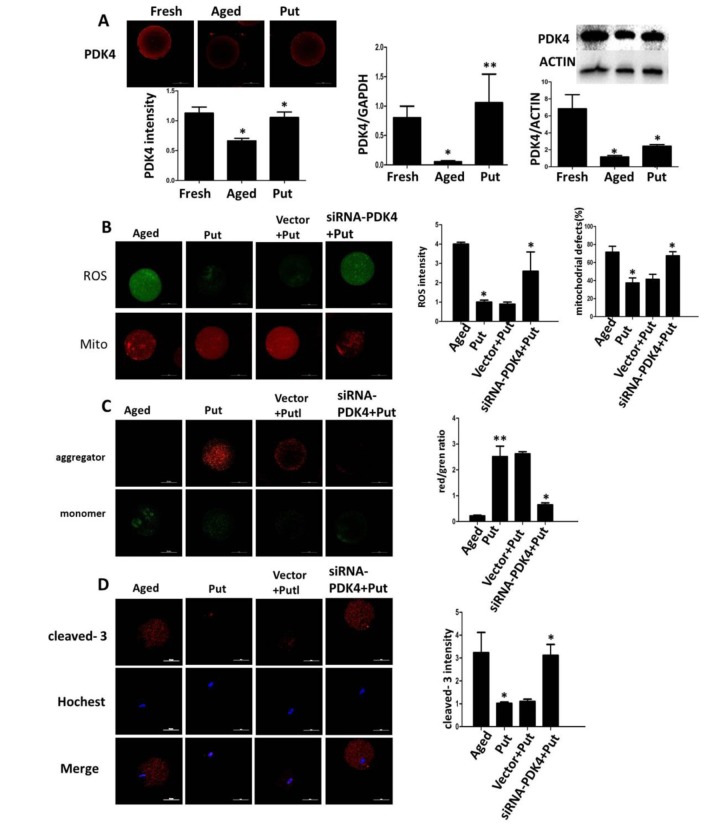
**Effects of putrescine on PDK4 expression, oxidative stress, and apoptosis in the oocytes during postovulatory aging.** (**A**) The effect of putrescine on PDK4 expression was tested by immunofluorescence, qPCR and Western blot. Putrescine rescued the downregulation of PDK4 at both the mRNA and protein levels in the aging oocytes. (**B**) The effects of putrescine on the ROS level and mitochondrial distribution in the aging oocytes. Putrescine significantly reduced the ROS level and the number of aggregated mitochondria in the aging oocytes. If PDK4 expression was downregulated by siRNA, the above effects of putrescine were significantly weakened. (**C**) The effect of putrescine on the MMP was related to mitochondrial activity. Putrescine significantly raised the MMP in the aging oocytes. However, this effect was blocked by siRNA-PDK4. (**D**) The effect of putrescine on the cleaved caspase 3 was related to apoptosis in the aging oocytes. Putrescine significantly inhibited the increased level of cleaved caspase 3 in the oocytes during postovulatory aging. This effect of putrescine was partially blocked by the downregulation of PDK4. *p<0.05, ** *p*<0.01.

We found that the expression of PDK4 was significantly downregulated in the postovulatory aging oocytes in the *in vitro* culture. The putrescine treatment partially rescued this effect of PDK4 expression (Figure 5A). To confirm the function of putrescine on the postovulatory aging of oocytes by upregulating PDK4, the expression of PDK4 was downregulated by injecting siRNA-PDK4 into fresh oocytes with or without putrescine in the medium for 24 h of *in vitro* culture. Then, the level of oxidative stress, the mitochondrial distribution and the MMP index and the apoptotic index were analyzed. We found that the ROS level and the caspase 3 activition were significantly increased in those oocytes with siRNA-PDK4 (Figure 5B). The defects in mitochondrial distribution were also increased (Figure 5B). Following the *in vitro* aging process, the MMP index was significantly reduced in the PDK4-downregulated oocytes that were treated synchronously with putrescine, when compared to the putrescine-treated oocytes (Figure 5C). Meanwhile, putrescine significantly inhibited the caspase 3 activation in the postovulatory aging oocytes, and this effect of putrescine was partially blocked by the downregulation of PDK4 (Figure 5D).

## DISCUSSION

**Figure 6:**
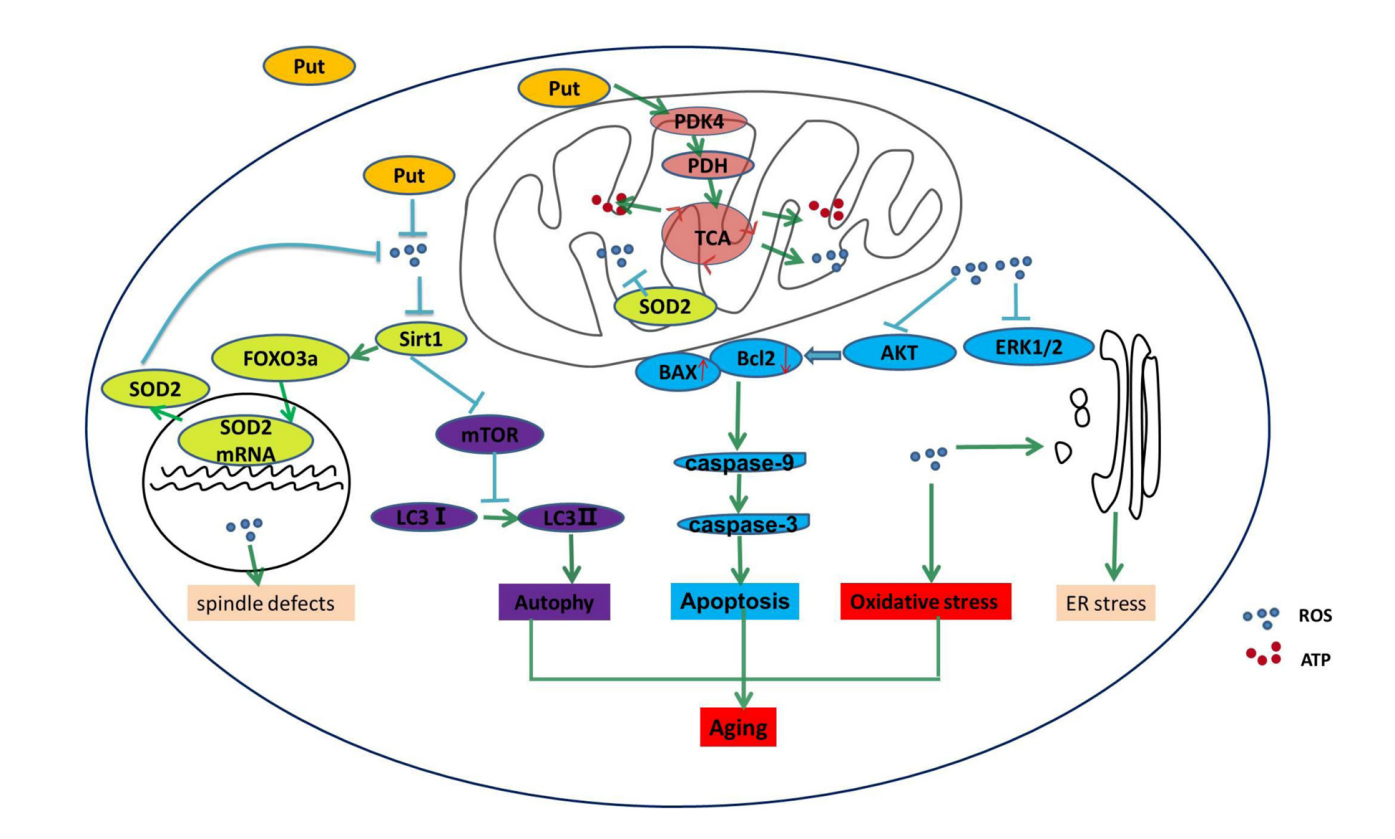
**Putrescine delayed the postovulatory aging of MII oocytes by regulating PDK4 and mitochondrial activity.** In the present study, we explored three key mechanisms in the oocytes during postovulatory aging, including oxidative stress, apoptosis, and mitochondrial autophagy. Putrescine exerts a protective role during the postovulatory aging process by regulating mitochondrial function in addition to providing an antioxidant effect. Oxidant stress, showing as the increased ROS, is main mechanism of the aging of postovulatory oocytes. The level of PDK4, which is a key factor of TCA in the mitochondria of the aging oocytes, was significantly upregulated by putrescine. The effects of putrescine were partially blocked by the downregulation of PDK4. In conclusion, putrescine delays the aging of oocytes by regulating PDK4 expression, antioxidation and mitochondrial activity.

In the present study, we found that PDK4 exerted a protective role in the oocytes that were undergoing *in vitro* the postovulatory aging, and we demonstrated that PDK4 delayed the aging process of oocytes by decreasing the ROS level. Mitochondrial PDK4 reduces oxidative stress, while putrescine delays the aging process in postovulatory oocytes by increasing the expression of PDK4 (Figure 6). Therefore, putrescine may be used as an antioxidant, a PDK4 activator or a protective factor in the *in vitro* culture of oocytes for basic research and the clinical practice of reproductive medicine.

Oxidative stress acts as a ‘trigger’ for a cascade of other factors that orchestrate the process of postovulatory aging *in vivo* and *in vitro*, and it directly affects multiple aspects of oocyte biochemistry and functionality [27]. There are more mitochondria in MII oocytes than in granule cells (GCs), suggesting that the function of oocytes is more dependent on energy metabolism and that more ROS exist in oocytes than GCs because both ATP and ROS are concomitantly produced in the mitochondria via the TCA cycle [28]. The increased oxidative stress in the postovulatory aging oocytes is due to a progressive increase in ROS production and the concomitant depletion of antioxidant protection, as well as environmental factors [4]. Our study showed the progressive accumulation of ROS during the postovulatory aging of oocytes.

The ROS accumulation is a key negative factor of oocyte quality [29]. Previous studies in somatic cells have provided strong evidence for the role of Sirt1 as a sensor of the cellular redox state and a protector against ROS and aging [30]. In other words, ROS reduces the expression of Sirt1, and the downregulation of Sirt1 further increases the level of ROS, which results in a malignant cycle [31]. Notably, we demonstrated in the present study that the Sirt1 expression was lowered in concomitance with ROS accumulation. Our data suggested that Sirt1 played a pivotal role in the adaptive protection against ROS in the postovulatory aging oocytes [24]. According to the literature, one of the mechanisms through which Sirt1 decreases ROS and therefore promotes cell survival is the deacetylation of FOXO3a, a transcription factor that activates the expression of SOD2 [32]. SOD2, one of the well-known mitochondrial SODs, is a member of the iron/manganese superoxide dismutase family. The expression of SOD2 was significantly decreased following the high level of oxidative stress [33]. Consistent with those findings, the significant decrease of FoxO3a expression was verified in the postovulatory aging oocytes (Figure 6). Moreover, Sirt1 was also an important factor of spindle organization and mitochondrial distribution and activity in mouse oocytes [24]. Herein, we found that the proportions of spindle defect and mitochondrial distribution defect were increased and the MMP index was reduced, further supporting our conclusion that Sirt1 protects oocytes from the aging progress of postovulatory oocytes (Figure 6).

Autophagy is an essential cellular event that degrades the degenerated proteins and organelles in order to recycle their components in the cytoplasm. Moreover, autophagy is essential for the preimplantation process of early embryo development in mammals. Tsukamoto et al. showed that the basal level of autophagy was upregulated in early mouse embryos after fertilization [34]. Our data showed that autophagy was decreased in the *in vitro* aging oocytes. Furthermore, Sirt1 has been shown to enhance autophagy by inhibiting the activity of mTOR [35,36]. In accordance with this finding, we identified the Sirt1-mTOR-LC3-autophagy pathway as being regulated by ROS, thereby preventing the pathogenesis of aging in the postovulatory oocytes.

The accumulation of ROS has been shown to activate the mitochondrial-related apoptotic pathway [4,5]. We found in the present study that the apoptosis induced by the ROS accumulation was significantly increased during the aging progress of postovulatory oocytes. In addition, the related pathways of the ROS-induced apoptosis were the decreasing activation of AKT and ERK1/2 in the postovulatory aging oocytes (Figure 6), which was similar to those in the somatic cells [38]. In our previous study, we found that the activation of AKT and ERK1/2 inhibited the mitochondrial-related apoptotic pathway in Leydig cells [37,38]. Our results in this study further confirmed that the ROS accumulation induced the mitochondrial-related apoptosis by inactivating AKT and ERK1/2 during the aging of postovulatory oocytes.

As mentioned above, PDK4 plays diverse roles in anti-oxidation in different cells regardless of its classical effect on cellular metabolism. Our previous study had shown that PDK4 was expressed in human ovaries [39]. However, the role of PDK4 in ovarian tissue was unclear. In the present study, we collected the oocytes undergoing the aging *in vitro* and examined the expression of PDK4, so as to explore its role in the aging of oocytes. The immunoblot analysis showed that the expression level of PDK4 was dramatically decreased while the ROS level was increased as the oocyte aged, suggesting that PDK4 may reduce the accumulation of ROS in the postovulatory aging oocytes. As shown in Figure 6, PDK4 can phosphorylate PDH and thereby inactivate the TAC cycle in which ATP and ROS are generated together.

To further confirm the mutual effects of ROS and PDK4 in the postovulatory oocytes, putrescine was supplemented in the culture medium for 24 h, and the level of ROS, mitochondrial function and PDK4 expression were measured. Interestingly, the treatment with putrescine substantially promoted the expression of PDK4 during the postovulatory aging process, suggesting that putrescine might act as the front line of defense against the ROS accumulation through PDK4. Consistent with this finding, we observed the significant increases in the ROS level, abnormalities in chromosomes and spindle organization, mitochondrial aggregation, and the decrease in mitochondrial function, following the knockdown of PDK4 expression. Additionally, the knockdown of PDK4 expression accelerated apoptosis through the mitochondrial-related apoptotic pathways, suggesting that putrescine played an anti-oxidant role and improved the quality of the oocytes by partially upregulating PDK4 expression. It has been reported that hypoxia-inducible factor-1α (HIF-1α), one of the major factors during hypoxia, can bind to the promoter of PDK4 gene, which was evaluated by luciferase; and that hypoxia can also induce the PDK4 expression via estrogen related receptor γ (ERRγ) to suppress glucose metabolism and accelerate vascular calcification [40]. Besides, the PDK4 expression was increased by peroxisome proliferator-activated receptor-a (PPARa) and WY-14,643 (a potent agonist for PPARa receptor) in starvation and diabetes [41]. In the present study, we found the crosstalk between PDK4 and Sirt1 in the aging oocytes. The intranuclear mechanism of the upregulating PDK4 expression is worthy of study in future.

In summary, we demonstrated that PDK4 prevented the multiple changes that are associated with the aging of postovulatory oocytes and apoptosis by the anti-oxidation mechanisms. Moreover, putrescine prevented the accumulation of ROS and apoptosis in addition to decreasing mitochondrial function by enhancing the role of PDK4. Our findings provide the rationale for PDK4 as a key and potential therapeutic target to ameliorate and preserve the competence of oocytes, and support the application of putrescine to slow the oxidative stress-mediated aging of oocytes and ovarian dysfunction.

## MATERIALS AND METHODS

### Antibodies and chemicals

All chemicals used in this study were purchased from Sigma-Aldrich unless otherwise stated. The rabbit polyoclonal anti-α-tubulin FITC-conjugated antibodies, the anti-p-mTOR, anti-mTOR, ani-Sirt1, anti-FOXO3a, anti-LC I/II, anti-p-AKT, anti-AKT, anti-p-ERK1/2 and anti-BCL2 antibodies were from Cell Signaling Technology (Beverly MA, USA). The rabbit polyclonal anti-PDK4 antibody was from Novus (Dallas, TA, USA). The mouse monoclonal anti-ACTIN antibody was from Sigma (St. Louis, MO, USA).

### Animal care and welfare

The animals used for gamete collections herein were treated in accordance with the guidelines for the care and use of laboratory animals that were approved by the Animal Care Committee of Nanjing Medical University.

### Oocyte collection and culture conditions

CD-1 female mice that were 8 weeks old were injected with 5 IU of pregnant mare serum gonadotropin (PMSG; Sigma, St Louis, MO). MII oocytes were obtained from the oviducts 14-16 h after the injection of 5 IU hCG, which was administered 48 h after the PMSG injection. The oocytes were released into HEPES-buffered M2 followed by treatment with 0.1% bovine testes hyaluronidase for 1 min to remove cumulus cells. The MII oocytes were thoroughly washed and transferred into 20 μl drops of M2 under paraffin oil at 37°C and in a humidified atmosphere containing 5% CO_2_. The *in vitro* aging was accomplished by extending the culture time of oocytes after their collection in M2 medium with or without 0.5 mM putrescine to 24 h. The dosage of putrescine was referenced by Liu *et al*. (2017) .

### Microinjection

The siRNAs were prepared as described previously [42]. Approximately 5-10 pl of siRNA were microinjected into the cytoplasm of oocytes using a Femto Jet microinjector (Eppendorf, Hamburg, Germany) with a Leica inverted microscope (DMIRB) that was equipped with a micromanipulator (Narishige, Tokyo, Japan). After injection, the oocytes were cultured in M2 medium at 37°C for 24 h in an atmosphere of 5% CO2 in air.

### TUNEL assay

DNA fragmentation was detected by the TUNEL method with the use of an in situ cell death kit (Roche Diagnostic Gmbh, Mannheim, Germany). The oocytes were first fixed in 4% paraformaldehyde dissolved in PBS (pH 7.4) for 30 min and subsequently permeabilized with 0.5% Triton X-100 for 30 min. The experimental groups and control groups were incubated at 37°C for 1 h in 25 μl of the TUNEL reaction mixture containing dUTP-fluorescein isothiocyanate (FITC), terminal deoxynucleotydil transferase (TdT) enzyme, and reaction buffer. After they were washed three times, the samples were observed using a fluorescence microscope.

### Immunofluorescence

The oocytes were fixed and permeabilized as described above. The oocytes were transferred into 5% normal goat serum for 1 h followed by incubation at 37°C. Then, the oocytes were incubated overnight at 4°C with the primary antibodies (1:50-1:100 dilution), followed by incubation with Alexa Fluor 594-conjugated secondary antibodies (1:1000, Thermo Fisher) for 1 h at room temperature. Hoechst 33342 (10 mg/ml in PBS) was used for the DNA counterstaining. To measure the immunofluorescence intensity, the signals from both control and experimental oocytes were acquired by performing the same immunostaining procedure and setting up the same parameters of confocal microscope. The data were analyzed by Image J software (National Institutes of Health, Bethesda, Maryland, USA).

### Detection of intracellular ROS level

To identify the oxidative stress/ROS levels in aged oocytes, 50-carboxy-20,70- difluorodihydrofluorescein diacetate (carboxy-DFFDA; Molecular Probes), a fluorescent probe capable of detecting powerful oxidants such as H2O2 and peroxynitrite, was utilized. The oocytes were incubated in a 10 μM solution of carboxy-DFFDA in the M2 medium for 30 min at 37°C under 5% CO2 gas. Oocytes were then washed three times in the M2 medium before they were mounted on a glass slide for microscopy.

### Detection of mitochondrial distribution

For the mitochondrial staining, oocytes were incubated for 30 min at 37°C in an M2 medium that was supplemented with 200 nM MitoTracker Red (Invitrogen, Carlsbad, CA, USA). Next, the oocytes were mounted on glass slides and examined under a laser scanning confocal microscope (Nikon C2 Plus, Tokyo, Japan). A pixel intensity value was calculated for each oocyte using ImageJ software (National Institutes of Health).

### JC-1 assay for mitochondrial activity

The membrane potential sensitive dye JC-1 (Molecular Probes, Eugene, Oregon, USA) was used to estimate the functional activity of mitochondria. A 2 mM stock solution was prepared in DMSO and kept at −20°C. Before use, the stock solution was diluted to 2 μM in M2. The oocytes were incubated with the working solution for 30 min at 37°C before the confocal observation. All of the oocytes were captured under the same exposure intensity. To calculate the confocal ratio of red to green images, the intensities of green and red fluorescence were quantified separately with Image J software.

### Western blotting

Cell lysates from 100 cumulus-free oocytes were prepared by adding 10 μl of 2X sample buffer (SB) (Laemmli 1970) as described previously [43]. The samples were boiled for 5 min and loaded onto 4–20% Tris-Acetate gels (Laemmli 1970), and the proteins were separated using electrophoresis and transferred onto PVDF membranes (Micron Separations, Westboro, MA). The membranes were blocked and then probed with specific anti-Sirt1 (1:500), anti-FOXO3a (1:500), anti-SOD2 (1:500), anti-LC I/II (1:500), anti-p-mTOR (1:500), anti-mTOR (1:1000), anti-p-AKT (1:500), anti-AKT (1:1000), anti-p-ERK1/2 (1:1000), anti-ERK1/2 (1:1000), anti-BCL2 and anti-ACTIN (1:1000) antibodies at 4°C overnight. After the membranes were incubated with HRP-conjugated secondary antibodies at room temperature for 1 h, an enhanced chemiluminescence (ECL) kit was used to detect the signals. The bands were semiquantified with the analysis software that was provided by the imaging system.

### Real-time RT-PCR analysis

Total RNA was extracted from 100 oocytes using a RNeasy Plus Micro Kit (Qiagen, Hilden, Germany). First-strand cDNA was generated using a cDNA synthesis kit (Qiagen, Hilden, Germany). GAPDH was selected as the reference gene. The primer sequences are as follows: PDK4 forward: 5’-TGATTGGCTACTGTAAAAGTCCCGC-3’; reverse: 5’-ATCCCAGGTCCCTAGGACTTCAGG-3’; SOD2 forward: 5’-GGGGGCCGGCCGAGAGCAGCGGT

CGTGTA-3’; reverse: 5’-GGGGGCGCGCCATGTGGCCGTGAGTGAG-3’. The SYBR® FAST qPCR Kits (Takara, Wilmington, MA, USA) were used in combination with a Real-Time PCR Detection System (A, Hercules, CA, USA). The relative gene expression was calculated by the 2^ΔΔCT^ method.

### Statistical analyses

Statistical analysis was performed using SPSS 17 Software. All of the experiments were repeated at least three times, and each experimental group included at least 20 oocytes unless otherwise specified. The significance of the differences between groups was analyzed by ANOVA. Data are expressed as the means ± SEM; differences with p values <0.05 were considered statistically significant.

## Supplementary Material

Supplementary Figure

## References

[1] Fissore RA, Kurokawa M, Knott J, Zhang M, Smyth J. Mechanisms underlying oocyte activation and postovulatory ageing. Reproduction. 2002; 124:745–54. 12530912 10.1530/rep.0.1240745

[2] Miao YL, Kikuchi K, Sun QY, Schatten H. Oocyte aging: cellular and molecular changes, developmental potential and reversal possibility. Hum Reprod Update. 2009; 15:573–85. 10.1093/humupd/dmp014 19429634

[3] Lord T, Aitken RJ. Oxidative stress and ageing of the post-ovulatory oocyte. Reproduction. 2013; 146:R217–27. 10.1530/REP-13-0111 23950493

[4] Lord T, Nixon B, Jones KT, Aitken RJ. Melatonin prevents postovulatory oocyte aging in the mouse and extends the window for optimal fertilization in vitro. Biol Reprod. 2013; 88:67. 10.1095/biolreprod.112.106450 23365415

[5] Tatone C, Carbone MC, Gallo R, Delle Monache S, Di Cola M, Alesse E, Amicarelli F. Age-associated changes in mouse oocytes during postovulatory in vitro culture: possible role for meiotic kinases and survival factor BCL2. Biol Reprod. 2006; 74:395–402. 10.1095/biolreprod.105.046169 16251501

[6] Prasad S, Koch B, Chaube SK. RO-3306 prevents postovulatory aging-mediated spontaneous exit from M-II arrest in rat eggs cultured in vitro. Biomed Pharmacother. 2016; 78:216–25. 10.1016/j.biopha.2016.01.013 26898445

[7] Zhang T, Zhou Y, Li L, Wang HH, Ma XS, Qian WP, Shen W, Schatten H, Sun QY. SIRT1, 2, 3 protect mouse oocytes from postovulatory aging. Aging (Albany NY). 2016; 8:685–96. 10.18632/aging.100911 26974211 PMC4925822

[8] Fukuda A, Roudebush WE, Thatcher SS. Influences of in vitro oocyte aging on microfertilization in the mouse with reference to zona hardening. J Assist Reprod Genet. 1992; 9:378–83. 10.1007/BF01203963 1472818

[9] Yanagida K, Yazawa H, Katayose H, Suzuki K, Hoshi K, Sato A. Influence of oocyte preincubation time on fertilization after intracytoplasmic sperm injection. Hum Reprod. 1998; 13:2223–26. 10.1093/humrep/13.8.2223 9756300

[10] Childs AC, Mehta DJ, Gerner EW. Polyamine-dependent gene expression. Cell Mol Life Sci. 2003; 60:1394–406. 10.1007/s00018-003-2332-4 12943227 PMC11138590

[11] Wallace HM, Fraser AV, Hughes A. A perspective of polyamine metabolism. Biochem J. 2003; 376:1–14. 10.1042/bj20031327 13678416 PMC1223767

[12] Biswas MS, Mano J. Lipid Peroxide-Derived Short-Chain Carbonyls Mediate Hydrogen Peroxide-Induced and Salt-Induced Programmed Cell Death in Plants. Plant Physiol. 2015; 168:885–98. 10.1104/pp.115.256834 26025050 PMC4741343

[13] Tanou G, Ziogas V, Belghazi M, Christou A, Filippou P, Job D, Fotopoulos V, Molassiotis A. Polyamines reprogram oxidative and nitrosative status and the proteome of citrus plants exposed to salinity stress. Plant Cell Environ. 2014; 37:864–85. 10.1111/pce.12204 24112028

[14] Kong X, Wang X, Yin Y, Li X, Gao H, Bazer FW, Wu G. Putrescine stimulates the mTOR signaling pathway and protein synthesis in porcine trophectoderm cells. Biol Reprod. 2014; 91:106. 10.1095/biolreprod.113.113977 25253735

[15] Liu D, Mo G, Tao Y, Wang H, Liu XJ. Putrescine supplementation during in vitro maturation of aged mouse oocytes improves the quality of blastocysts. Reprod Fertil Dev. 2017; 29:1392–400. 10.1071/RD16061 27319359

[16] Roche TE, Hiromasa Y. Pyruvate dehydrogenase kinase regulatory mechanisms and inhibition in treating diabetes, heart ischemia, and cancer. Cell Mol Life Sci. 2007; 64:830–49. 10.1007/s00018-007-6380-z 17310282 PMC11136253

[17] Behal RH, Buxton DB, Robertson JG, Olson MS. Regulation of the pyruvate dehydrogenase multienzyme complex. Annu Rev Nutr. 1993; 13:497–520. 10.1146/annurev.nu.13.070193.002433 8369156

[18] Liu Z, Chen X, Wang Y, Peng H, Wang Y, Jing Y, Zhang H. PDK4 protein promotes tumorigenesis through activation of cAMP-response element-binding protein (CREB)-Ras homolog enriched in brain (RHEB)-mTORC1 signaling cascade. J Biol Chem. 2014; 289:29739–49. 10.1074/jbc.M114.584821 25164809 PMC4207987

[19] Liu LX, Rowe GC, Yang S, Li J, Damilano F, Chan MC, Lu W, Jang C, Wada S, Morley M, Hesse M, Fleischmann BK, Rabinowitz JD, et al. PDK4 Inhibits Cardiac Pyruvate Oxidation in Late Pregnancy. Circ Res. 2017; 121:1370–78. 10.1161/CIRCRESAHA.117.311456 28928113 PMC5722682

[20] Wu J, Zhao Y, Park YK, Lee JY, Gao L, Zhao J, Wang L. Loss of PDK4 switches the hepatic NF-κB/TNF pathway from pro-survival to pro-apoptosis. Hepatology. 2018; 68:1111–24. 10.1002/hep.29902 29603325 PMC6165716

[21] Liu M, Yin Y, Ye X, Zeng M, Zhao Q, Keefe DL, Liu L. Resveratrol protects against age-associated infertility in mice. Hum Reprod. 2013; 28:707–17. 10.1093/humrep/des437 23293221

[22] Lord T, Martin JH, Aitken RJ. Accumulation of electrophilic aldehydes during postovulatory aging of mouse oocytes causes reduced fertility, oxidative stress, and apoptosis. Biol Reprod. 2015; 92:33. 10.1095/biolreprod.114.122820 25505195

[23] Brunet A, Sweeney LB, Sturgill JF, Chua KF, Greer PL, Lin Y, Tran H, Ross SE, Mostoslavsky R, Cohen HY, Hu LS, Cheng HL, Jedrychowski MP, et al. Stress-dependent regulation of FOXO transcription factors by the SIRT1 deacetylase. Science. 2004; 303:2011–15. 10.1126/science.1094637 14976264

[24] Di Emidio G, Falone S, Vitti M, D’Alessandro AM, Vento M, Di Pietro C, Amicarelli F, Tatone C. SIRT1 signalling protects mouse oocytes against oxidative stress and is deregulated during aging. Hum Reprod. 2014; 29:2006–17. 10.1093/humrep/deu160 24963165

[25] Ma R, Zhang Y, Zhang L, Han J, Rui R. Sirt1 protects pig oocyte against in vitro aging. Anim Sci J. 2015; 86:826–32. 10.1111/asj.12360 25601632

[26] Zhou XL, Xu JJ, Ni YH, Chen XC, Zhang HX, Zhang XM, Liu WJ, Luo LL, Fu YC. SIRT1 activator (SRT1720) improves the follicle reserve and prolongs the ovarian lifespan of diet-induced obesity in female mice via activating SIRT1 and suppressing mTOR signaling. J Ovarian Res. 2014; 7:97. 10.1186/s13048-014-0097-z 25330910 PMC4232623

[27] Zhang CX, Cui W, Zhang M, Zhang J, Wang TY, Zhu J, Jiao GZ, Tan JH. Role of Na+/Ca2+ exchanger (NCX) in modulating postovulatory aging of mouse and rat oocytes. PLoS One. 2014; 9:e93446. 10.1371/journal.pone.0093446 24695407 PMC3973580

[28] May-Panloup P, Boucret L, Chao de la Barca JM, Desquiret-Dumas V, Ferré-L’Hotellier V, Morinière C, Descamps P, Procaccio V, Reynier P. Ovarian ageing: the role of mitochondria in oocytes and follicles. Hum Reprod Update. 2016; 22:725–43. 10.1093/humupd/dmw028 27562289

[29] Takahashi T, Takahashi E, Igarashi H, Tezuka N, Kurachi H. Impact of oxidative stress in aged mouse oocytes on calcium oscillations at fertilization. Mol Reprod Dev. 2003; 66:143–52. 10.1002/mrd.10341 12950101

[30] Salminen A, Kaarniranta K, Kauppinen A. Crosstalk between Oxidative Stress and SIRT1: Impact on the Aging Process. Int J Mol Sci. 2013; 14:3834–59. 10.3390/ijms14023834 23434668 PMC3588074

[31] Caito S, Rajendrasozhan S, Cook S, Chung S, Yao H, Friedman AE, Brookes PS, Rahman I. SIRT1 is a redox-sensitive deacetylase that is post-translationally modified by oxidants and carbonyl stress. FASEB J. 2010; 24:3145–59. 10.1096/fj.09-151308 20385619 PMC2923349

[32] Yan Z, Dai Y, Fu H, Zheng Y, Bao D, Yin Y, Chen Q, Nie X, Hao Q, Hou D, Cui Y. Curcumin exerts a protective effect against premature ovarian failure in mice. J Mol Endocrinol. 2018; 60:261–71. 10.1530/JME-17-0214 29437881 PMC5863768

[33] Hung CH, Chan SH, Chu PM, Tsai KL. Quercetin is a potent anti-atherosclerotic compound by activation of SIRT1 signaling under oxLDL stimulation. Mol Nutr Food Res. 2015; 59:1905–17. 10.1002/mnfr.201500144 26202455

[34] Tsukamoto S, Kuma A, Murakami M, Kishi C, Yamamoto A, Mizushima N. Autophagy is essential for preimplantation development of mouse embryos. Science. 2008; 321:117–20. 10.1126/science.1154822 18599786

[35] Gurusamy N, Lekli I, Mukherjee S, Ray D, Ahsan MK, Gherghiceanu M, Popescu LM, Das DK. Cardioprotection by resveratrol: a novel mechanism via autophagy involving the mTORC2 pathway. Cardiovasc Res. 2010; 86:103–12. 10.1093/cvr/cvp384 19959541 PMC2836260

[36] Sugiyama M, Kawahara-Miki R, Kawana H, Shirasuna K, Kuwayama T, Iwata H. Resveratrol-induced mitochondrial synthesis and autophagy in oocytes derived from early antral follicles of aged cows. J Reprod Dev. 2015; 61:251–59. 10.1262/jrd.2015-001 25866375 PMC4547982

[37] Xu W, Zhu Q, Zhang B, Liu S, Dai X, Gao C, Gao L, Cui Y. Protective effect of calretinin on testicular Leydig cells via the inhibition of apoptosis. Aging (Albany NY). 2017; 9:1269–79. 10.18632/aging.101226 28437248 PMC5425126

[38] Palanivel K, Kanimozhi V, Kadalmani B, Akbarsha MA. Verrucarin A induces apoptosis through ROS-mediated EGFR/MAPK/Akt signaling pathways in MDA-MB-231 breast cancer cells. J Cell Biochem. 2014; 115:2022–32. 10.1002/jcb.24874 24963595

[39] Diao FY, Xu M, Hu Y, Li J, Xu Z, Lin M, Wang L, Zhou Y, Zhou Z, Liu J, Sha J. The molecular characteristics of polycystic ovary syndrome (PCOS) ovary defined by human ovary cDNA microarray. J Mol Endocrinol. 2004; 33:59–72. 15291743 10.1677/jme.0.0330059

[40] Zhu Y, Ma WQ, Han XQ, Wang Y, Wang X, Liu NF. Advanced glycation end products accelerate calcification in VSMCs through HIF-1α/PDK4 activation and suppress glucose metabolism. Sci Rep. 2018; 8:13730. 10.1038/s41598-018-31877-6 30213959 PMC6137084

[41] Huang B, Wu P, Bowker-Kinley MM, Harris RA. Regulation of pyruvate dehydrogenase kinase expression by peroxisome proliferator-activated receptor-alpha ligands, glucocorticoids, and insulin. Diabetes. 2002; 51:276–83. 10.2337/diabetes.51.2.276 11812733

[42] Jeon HJ, Cui XS, Guo J, Lee JM, Kim JS, Oh JS. TCTP regulates spindle assembly during postovulatory aging and prevents deterioration in mouse oocyte quality. Biochim Biophys Acta Mol Cell Res. 2017; 1864:1328–34. 10.1016/j.bbamcr.2017.05.002 28476647

[43] Xu W, Zhu Q, Liu S, Dai X, Zhang B, Gao C, Gao L, Liu J, Cui Y. Calretinin Participates in Regulating Steroidogenesis by PLC-Ca^2+^-PKC Pathway in Leydig Cells. Sci Rep. 2018; 8:7403. 10.1038/s41598-018-25427-3 29743498 PMC5943404

