# Supplementary material for: Putrescine delays postovulatory aging of mouse oocytes by upregulating PDK4 expression and improving mitochondrial activity
Source: Aging (Albany NY). 2018 Dec 16;10(12):4093–106. doi: 10.18632/aging.101699 (PMC6326651; doi:10.18632/aging.101699)
Supplement: Supplementary Figure [file aging-10-101699-s001.pdf]

SUPPLEMENTARY FIGURE

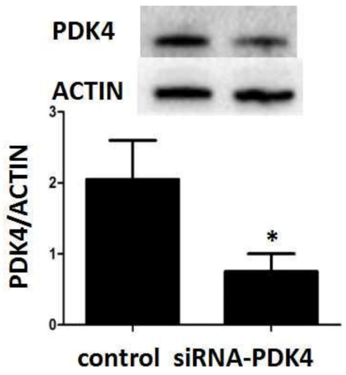

Supplementary Figure 1. PDK4 expression was downregulated after siRNA-PDK4 was injected into oocytes. The siRNAs were prepared as described previously [21]. Approximately 5–10 pl of siRNA were microinjected into the cytoplasm of oocytes using a FemtoJet microinjector (Eppendorf, Hamburg, Germany) with a Leica inverted microscope (DMIRB) equipped with a micromanipulator (Narishige, Tokyo, Japan). After injection, oocytes were cultured in M16 medium at 37°C for 24 h in an atmosphere of 5% CO_2. Compared with the control, PDK4 expression was significantly downregulated after siRNA-PDK4 infection. The inhibition rate of PDK4 expression was approximately 65%. *: $p<0.05$.
